# The Protective Effect of Beraprost Sodium on Diabetic Nephropathy by Inhibiting Inflammation and p38 MAPK Signaling Pathway in High-Fat Diet/Streptozotocin-Induced Diabetic Rats

**DOI:** 10.1155/2016/1690474

**Published:** 2016-04-24

**Authors:** Li Peng, Jie Li, Yixing Xu, Yangtian Wang, Hong Du, Jiaqing Shao, Zhimin Liu

**Affiliations:** ^1^Department of Endocrinology, Nanjing General Hospital of Nanjing Military Command (Jinling Hospital), 305 East Zhongshan Road, Nanjing, Jiangsu 210002, China; ^2^Department of Endocrinology, Shanghai Changzheng Hospital, Second Military Medical University, 415 Fengyang Road, Shanghai 200003, China

## Abstract

*Background*. p38 mitogen-activated protein kinase (MAPK) plays a crucial role in regulating signaling pathways implicated in inflammatory processes leading to diabetic nephropathy (DN). This study aimed to examine p38 MAPK activation in DN and determine whether beraprost sodium (BPS) ameliorates DN by inhibiting inflammation and p38 MAPK signaling pathway in diabetic rats.* Methods*. Forty male Sprague Dawley (SD) rats were randomly divided into the normal control group, type 2 diabetic group, and BPS treatment group. At the end of the 8-week experiment, we measured renal pathological changes and the activation of the p38 MAPK signaling pathway and inflammation.* Result*. After BPS treatment, renal function, 24-hour urine protein, lipid profiles, and blood glucose level were improved significantly; meanwhile, inflammation and the expression of p38 MAPK signaling pathway in the diabetic kidney were attenuated.* Conclusions*. BPS significantly prevented type 2 diabetes induced kidney injury characterized by renal dysfunction and pathological changes. The protective mechanisms are complicated but may be mainly attributed to the inhibition of the p38 MAPK signaling pathway and inflammation in the diabetic kidney.

## 1. Introduction

Diabetic nephropathy (DN) is a leading cause of end-stage renal failure, accounting for 35–40% of all new cases requiring dialysis therapy throughout the world [1]. The increasing incidence of diabetes elevates DN to one of the most important current public health issues, representing a significant burden on the health system. Strict control of blood glucose and blood pressure levels sometimes fails to delay the development of DN, and an effective therapy is not yet available [2, 3]. We need more innovative strategies to prevent and treat this disease. A comprehensive understanding of the mechanisms of DN is essential to develop new strategies.

Mitogen-activated protein kinases (MAPKs) are a large family of ubiquitously expressed protein serine/threonine kinases that respond to a variety of extracellular stimuli and mediate intracellular signal transduction [4]. In recent years, a series of studies have identified association between high glucose and activation of MAPKs in the development of chronic complications in diabetes [5, 6]. Activation of the MAPKs pathway induces enhanced extracellular matrix protein and Transforming Growth Factor-*β*1 (TGF-*β*1) expression, suggesting that activation of the MAPK signal transduction pathways might be responsible for abnormalities in diabetic glomeruli, leading to the development of DN [7].

The p38 group of MAP kinases is one of the four main subgroups in MAPKs family, serving as a nexus for signal transduction, and play a vital role in numerous biological processes [8]. The activation of p38 MAPK signaling pathway has been observed in response to a variety of extracellular stimuli such as UV light, heat, osmotic shock, inflammatory cytokines (TNF-*α*  and IL-1), and growth factors (CSF-1) in different organisms [9]. More evidences show the links between p38 MAPK and inflammation, cell cycle, cell death, development, cell differentiation, senescence, and tumorigenesis in specific cell types [8]. Recent studies have shown that inflammation is associated with the development and progression of DN, implying that inflammatory mechanisms may play a pivotal role in the disease process [10, 11]. The p38 MAPK pathway plays a major role in the regulation of inflammatory responses [12]. Activation of the p38 MAPK pathway can promote renal inflammation. Therefore, the p38 MAPK signaling pathway and inflammation may be an important pathogenic mechanism underlying DN. Recent in vitro studies have shown that high levels of glucose can activate the p38 MAPK signaling pathway in renal cells and induce the phosphorylation of p38 MAPK and inflammatory cytokine production, which promotes the production of Fibronectin by the mesangial cells [13]. There is a wealth of data that supports the central role of the p38 MAPK signaling pathway in high glucose-induced cell damage and activation of inflammation [14]. Therefore, it has been proposed that inhibition of the p38 MAPK signaling pathway may reduce the formation of the extracellular matrix (ECM) in the glomerular mesangium and block the thickening of the glomerular basement membrane [15], thus preventing the development of DN.

Beraprost sodium (BPS) is a new stable, orally active prostaglandin I_2_ analogue with antiplatelet and vasodilating properties [16]. There is ample evidence to link PGI2 signaling to individual cell processes contributing to progressive kidney injury, including alterations in renal growth, fibrosis/sclerosis, and apoptosis [17]. Some studies have reported that beraprost sodium (BPS) is a vasoactive substance that can expand renal vessels, increase renal blood flow, inhibit TXA2 synthesis, platelet aggregation, and immune complex formation, and prevent glomerular thrombosis to finally reduce proteinuria [2]. In obese Zucker rats, BPS can suppress the pathogenesis and development of diabetes and its complication, nephropathy, which was presumably accompanied by improving glucose intolerance and insulin resistance [18]. For these reasons, BPS may be highly effective for the prevention and treatment of microvascular complications of diabetes mellitus (DM). Our previous studies have shown that high concentrations of glucose can stimulate the overproduction of inflammatory cytokine and the expression of ECM and p38 MAPK in glomerular mesangial cells [19, 20]. However, whether BPS can mitigate high glucose-induced renal glomerular mesangial cell proliferation, as well as the mechanisms underlying the action of BPS, has not been explored.

DN is characterized by glomerular hypertrophy, excessive accumulation of ECM components, sclerosis, and end-stage interstitial fibrosis. Notably, inflammation has been thought to play a predominant role in the pathogenesis of progressive renal disease. Therefore, the discovery of new strategies to inhibit inflammation and delay mesangial cell proliferation and ECM accumulation in the glomeruli will be of great significance for treating diabetic nephropathy in the clinical setting.

Currently, it is believed that p38 MAPK is an important signal transducer in DN, and inhibition of the p38 MAPK pathway may represent a new therapeutic target for preventing the development of DN. However, the specific mechanism remains to be elucidated. Some studies demonstrate that receptor-specific cross talk between the p38 MAPK and PGI2 pathways may regulate the extent of prostanoid synthesis in human endothelial cells [21]. In this study, we examined the effects of BPS treatment on streptozotocin-induced diabetic rats fed with high-fat diet (HFD), a type 2 DM animal model, and investigated the mechanisms underlying the protective action of BPS in DN induced by inflammation and activation of p38 signaling pathway; in addition, we explored whether the protective effects were mediated by inhibition of the p38 MAPK signaling pathway.

## 2. Materials and Methods

### 2.1. Reagents

Streptozotocin (STZ) was purchased from Sigma Chemicals Company (St. Louis, MO, USA). BPS (Dena) used in this study was a gift from Astellas Pharma Inc. Blood glucose, blood urea nitrogen (BUN), serum creatinine, blood lipid profile (TC, TG, HDL, and LDL), SOD, MDA, GSH, IL-6, TNF-*α*, MPO, hs-CRP, NO, ET-1, and urinary albumin assay kits were purchased from Nanjing Jiancheng Biotech (Nanjing, China). The mouse anti-human p-p38 antibody, mouse anti-human total p38 (t-p38) antibody, goat anti-human TNF-*α* antibody, goat anti-human TGF-*β*1 antibody, goat anti-human MMP-9 antibody, goat anti-rat COX-2 antibody, goat anti-rat FN antibody, goat anti-human CREB antibody, mouse anti-*β*-actin, immunohistochemistry kits for collagen type IV, and CD31 were from Santa Cruz Biotechnology (USA). DEPC was provided by Shanghai Biocolor BioScience & Technology. Trizol, the RT-PCR reagent kit, and DNA markers were from Invitrogen. Primers for p38 MAPK target genes and the internal control GAPDH were synthesized by Shanghai DaWeiKe Biotechnology. The RNA Guard reagent was purchased from Shanghai Huashun Biological Reagent Co. The ReverTra Ace^TM^ reverse transcription reagent kit and SYBR Green Real-Time PCR Master Mix were from TOYOBO (Japan). The total cellular protein extraction reagent was from KeyGEN Biotech (Nanjing). Citric acid and sodium citrate were purchased from Sinopharm Chemical Reagent Co, Ltd. 2.5% glutaraldehyde for scanning electron microscopy was purchased from Fudan University's School of Medicine. 4% paraformaldehyde was from Department of Pathology, Affiliated Changzheng Hospital, Second Military Medical University. 10% chloral hydrate was from Second Military Medical University Experimental Animal Center.

### 2.2. Animals and Experimental Protocols

Forty 6-week-old specific pathogen-free (SPF) grade male Sprague Dawley (SD) rats weighing approximately 180 ± 20 g were purchased from the Animal Center of the Second Military Medical University. The animal production permission was SCXK (Shanghai) 2007-0003, and the animal use permission was SYXK (Shanghai) 2007-0003. Animals were housed in clean-grade animal rooms at the Experimental Animal Center of the Second Military Medical University. They were maintained under standard conditions of temperature (21 ± 2°C) and humidity (55 ± 2%) with an alternating 12 h light/dark cycles. The animals had free access to tap water and were fed with adequate food. All the experiments with animals were carried out according to the principles of experimental animal care (NIH Publication number 85-23, amended in 1985).

After 1 week of adaptive feeding, the rats were randomly divided into model group (*n* = 15), BPS group (*n* = 15), and normal control group (*n* = 10) based on the random number table method. Both model and BPS group were fed with high-fat diet (HFD, 40% of calories from fat) for 4 weeks to establish the insulin-resistant model. Type 2 diabetes model was induced by intraperitoneal injection of a single dose streptozotocin (STZ, 30 mg/kg dissolved in 0.1 mol/L citric acid buffer, pH 4.3) to the insulin-resistant model rats. The rats in normal group were fed with normal chow (4% calories from fat) for 4 weeks and intraperitoneally injected with the same dose of citric acid buffer (pH 4.3, 0.1 mol/L). Blood glucose levels were measured on the third day after streptozotocin injection to confirm the development of diabetes. The blood glucose levels above 16.7 mmol/L after STZ injection were considered as diabetic. There were 24 rats in the successful T2DM model. Rats in BPS group were orally given 0.6 mg/kg/day of BPS. The rats in both model group and control group were intragastrically given an equal volume of double distilled water at the same time every day. The experiment lasted for 12 weeks (week 0–week 12). The body weight and blood glucose levels of rats were monitored every week. Animals were sacrificed at the end of week 12. At the end of the experiment, 6 animals were included in each experimental group.

### 2.3. Collection of Tissue Samples

Urine was collected from the rats housed in metabolic cages for 24 h. All the animals were killed (anesthetized by intraperitoneal injection of 10% chloral hydrate at 4 mg/kg) at the end of the 12th week. The body weight and weight of left kidney (H110-type analytic balance from Sartorius, Germany) were measured and evaluated between groups. Blood samples were obtained by puncturing the abdominal aorta at the time of sacrifice. Blood samples were placed in tubes with EDTA anticoagulant and centrifuged at −20°C for subsequent usage. Meanwhile, kidney samples were rapidly excised, weighed, and frozen in liquid nitrogen or fixed in 10% neutral-buffered formalin. The kidney weight-to-body weight ratio was calculated.

### 2.4. Measurement of Biochemical Indicators and Inflammatory Cytokines

Blood samples of rats were centrifuged at 3,000 rpm for 15 min at 4°C and the supernatant was collected for measurement of blood glucose (BG), blood urea nitrogen (BUN), serum creatinine (Scr), triglyceride (TG), total cholesterol (TC), high-density lipoprotein (HDL), and low-density lipoprotein (LDL) by using a Hitachi Model 7020 Series Automatic Biochemistry Analyzer (Japan). The serum levels of interleukin-6 (IL-6), high-sensitivity C-reactive protein (hs-CRP), Tumor Necrosis Factor-*α* (TNF-*α*), and endothelin-1 (ET-1) were measured by the double-bond Sandwich ELISA method. The serum level of superoxide dismutase (SOD) activity was assessed by measuring the dismutation of superoxide radicals generated by xanthine oxidase and hypoxanthine using a superoxide dismutase assay kit. The serum level of malondialdehyde (MDA) was assessed by Thiobarbituric Acid (TBA) assay. The serum levels of glutathione (GSH) and myeloperoxidase (MPO) activity were measured using colorimetric detection kit. The serum level of nitric oxide (NO) was measured using NADPH dependent nitrate reductases. For the quantification of urinary albumin, urine samples were separated by 10% SDS-PAGE, followed by Coomassie brilliant blue staining.

### 2.5. Histopathology and Ultrastructure of the Kidney

For histological assessments, small segments of renal cortex from the normal and experimental rats were fixed in 10% buffered formalin and were processed for paraffin sectioning. Sections of about 5 mm width were stained with hematoxylin-eosin (H&E) and Masson trichrome for assessment under light microscope (Olympus CX41 Phase Contrast Microscope, Japan). For electron microscopy (Philips CM120 TEM at 80 kV, Netherlands), renal cortex tubular tissues were fixed with 2.5% glutaraldehyde for 2 h and 1% osmium tetroxide for 2 h. The sample was dehydrated with a series of ethanol and acetone for 10 minutes, embedded in epoxy resin, and cut into ultrathin sections.

### 2.6. Immunohistochemical Staining of CD31 and Collagen Type IV in Kidneys

Immunostaining was performed on 4 *μ*m thick sections using renal cortex tissue after deparaffinization. Microwave antigen retrieval was performed in citrate buffer at pH 6.0 for 10 min prior to peroxide quenching with 3% H_2_O_2_ buffer for 10 min. Sections were washed in water and perblocked with normal goat serum for 10 min. After blocking, the sections were incubated with mouse CD31 and collagen IV antibody at 4°C, followed by biotinylated anti-mouse IgG secondary antibody for 30 min at 37°C. Labeling was visualized with chromogen diaminobenzidine (DAB) and sections were counterstained with hematoxylin. Coverslips were mounted with Permount mounting solution. At last 10 random fields of each section were examined at 200x, which were analyzed by a computer image analysis system (IHC-image analysis from Shanghai DaWeiKe Biotechnology). In each field the value of the positive integrated optical density (IOD) was calculated, comparing the mean value.

### 2.7. Detection of p38 MAPK Gene Expression in Kidney Using RT-PCR (Table 1)

Primers for p38 MAPK target genes and the internal control GAPDH were synthesized by Shanghai DaWeiKe Biotechnology (primer sequences are shown in Table 1).

The total cellular protein extraction reagent was from KeyGEN Biotech (Nanjing). Reverse transcription was carried out with 1 *μ*g total RNA using SuperScript III (Invitrogen). Real-time PCR was performed applying a standard two-step amplification protocol on ABI 7500 system (Applied Biosystems) to detect mRNA expression. Each sample of renal cortex tissue was repeated in 6 wells, and a negative control without template cDNA was also used. The amplification conditions were 50°C for 2 min; 95°C for 10 min; and 40 cycles of 95°C for 15 s and 60°C for 1 min. After amplification, the melting curve was plotted starting from 60°C to validate the specificity of the amplification products. Relative expression values were obtained by normalizing C_T_ values of the tested genes to the C_T_ values of the housekeeping gene GAPDH using the ΔΔC_T_ method [22].

### 2.8. Detection of p38 MAPK Signaling Pathway Activation in Renal Tissues and the Protein Levels of TNF-*α*, TGF-*β*1, MMP-9, COX-2, CREB, and FN Using Western Blotting

One hundred micrograms of rat renal cortex tissue was weighed on ice and stored at −80°C after being aliquoted. Protein samples were quantitated using the BCA method; the absorbance of each well was determined using a microplate reader at a wavelength of 560 nm, and a standard curve was plotted. *β*-actin was used as a loading control in Western blot analysis. An 8% resolving gel with a 4% stacking gel was prepared, and samples were loaded for electrophoresis. After protein samples were transferred onto a membrane and blocked for approximately 1 h, 1 : 1000 dilutions of the primary antibodies, t-p38 MAPK, p-p38 MAPK, Tumor Necrosis Factor-*α* (TNF-*α*), Transforming Growth Factor-*β*1 (TGF-*β*1), Matrix Metalloprotein-9 (MMP-9), Cyclooxygenase-2 (COX-2), cAMP-response element binding protein (CREB), or Fibronectin (FN), were added and incubated at 4°C overnight. Horseradish peroxidase- (HRP-) conjugated secondary antibodies at 1 : 2000 dilution were added and incubated at 37°C for 1.5 h. After washing with TBST four times for 10 min, protein bands were analyzed using the Bio Image System (a gel documentation system, Syngene, A Division of Synoptic, Ltd.) to obtain optical density values.

### 2.9. Statistical Analysis

Statistical analyses were performed using the SPSS for Windows 13.0 software. Count data are presented as ($\overset{-}{X}\pm S$). The comparison of mean values among multiple groups was performed using the one-way analysis of variance (ANOVA); the comparison between two groups was examined using the least significant difference (LSD) test. The comparison of physiological and metabolic indicators before and after drug administration was performed using the paired *t*-test. *P* < 0.05 indicated that the difference was statistically significant.

## 3. Results

### 3.1. Effect of BPS on the Body Weight, Kidney Weight, Blood Glucose, and Blood Lipid Profile (TC, TG, HDL, and LDL) of HFD/STZ-Induced Diabetic Rats

**Figure 1:**
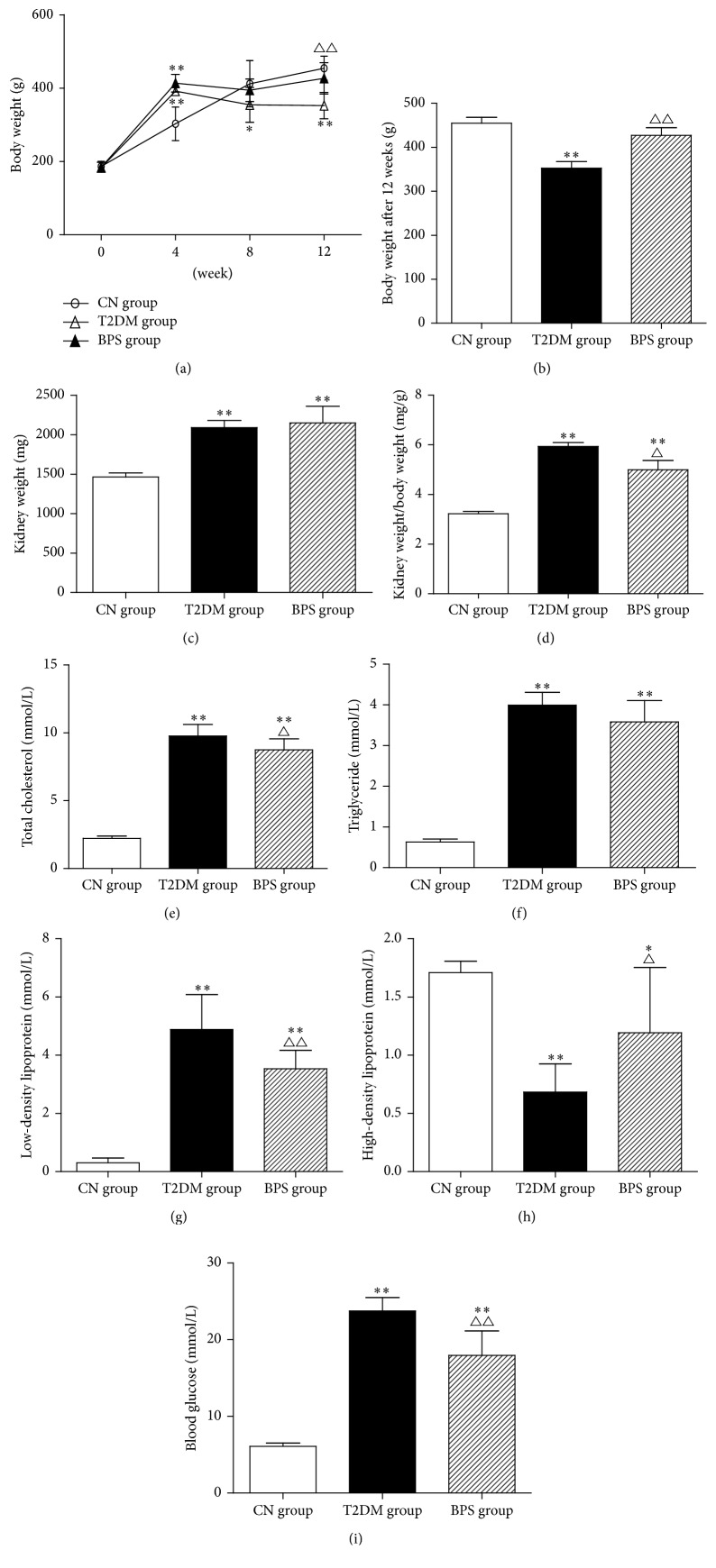
Body weight (a), body weight after 12 weeks (b), kidney weight (c), kidney weight/body weight (d), TC (e), TG (f), LDL-C (g), HDL-C (h), and blood glucose (i) in CN group rats, T2DM group rats, and BPS group rats. As line graph and bar graph, data are mean ± SEM of 6 rats. ^*∗*^ *P* < 0.05 and ^*∗∗*^ *P* < 0.01 T2DM group and BPS group versus CN group rats and ^∆^ *P* < 0.05 and ^∆∆^ *P* < 0.01 BPS group versus T2DM group.

HFD/STZ-induced rats in T2DM group characterized by the significant increase in blood glucose level (23.75 ± 1.75 mmol/L) and treated with 0.6 mg/kg BPS exhibited significantly higher blood glucose levels than rats in CN group (6.1 ± 0.43 mmol/L), but blood glucose of rats in PBS group was significantly lower (17.96 ± 3.19 mmol/L) than that of rats in T2DM group until the end of experiment (Figure 1(i), Table 3).

Body weight of rats in T2DM group and BPS group was significantly greater than that of rats in CN group at 4 weeks of experiment. During the experiment period, body weight of rats in CN group steadily increased. In comparison, body weight of rats in T2DM group and BPS group significantly decreased after 4 weeks of experiment (Figure 1(a), Table 2). At the end of experiment, body weight of rats in BPS group was significantly higher than that ot rats in T2DM group but lower than that of rats in CN group (Figure 1(b), Table 3). Posttreatment with BPS for 12 weeks after STZ exposure decreased the blood glucose levels and reduced the body weight loss, suggesting BPS's role as a good agent of antihyperglycemic nature and improvement of metabolism in HFD/STZ-induced type 2 diabetic rats.

In the HFD/STZ-induced diabetic rats, animals gained kidney weight and the kidney-to-body weight ratio (a marker for the development of diabetic nephropathy) was also increased (Figures 1(c) and 1(d), Table 3), compared to CN group. In BPS group, the kidney-to-body weight ratio was lower compared to rats in T2DM group.

Regarding the parameters related to lipid metabolism, TG levels were significantly higher in rats of T2DM group and BPS group, compared with rats in CN group at the end of experiment (Figure 1(f), Table 3). TC levels in T2DM group and BPS group were significantly greater than that in CN group. However, compared with the T2DM group, TC levels were significantly lower in BPS group (Figure 1(e), Table 3). Similarly, serum LDL-C was significantly higher in rats of T2DM group and BPS group than that in CN group until the end of experiment. Significantly lower LDL-C levels were observed in BPS group, compared with T2DM group (Figure 1(g), Table 3). Serum HDL-C was significantly lower in rats of T2DM group and BPS group than that in CN group until the end of experiment. Significantly higher HDL-C levels were observed in BPS group, compared with T2DM group (Figure 1(h), Table 3).

### 3.2. Effects of BPS on Kidney Function and 24 h Urinary Albumin of HFD/STZ-Induced Diabetic Rats

**Figure 2:**
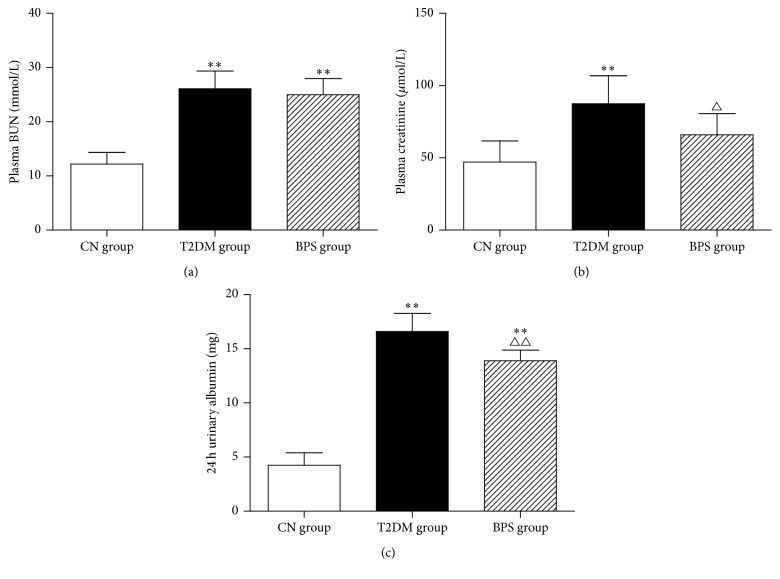
Plasma BUN (a), plasma creatinine (b), and 24 h urinary albumin (c) in CN group rats, T2DM group rats, and BPS group rats. As bar graph, data are mean ± SEM of 6 rats. ^*∗*^ *P* < 0.05 and ^*∗∗*^ *P* < 0.01 T2DM group and BPS group versus CN group rats and ^∆^ *P* < 0.05 and ^∆∆^ *P* < 0.01 BPS group versus T2DM group.

Plasma BUN, plasma creatinine, and 24 h urinary albumin showed significant elevation in rats of T2DM and BPS group, compared with CN group. However, BPS treatment efficiently reduced plasma creatinine and 24 h urinary albumin, acting as a nephroprotective agent in diabetes (Figures 2(a), 2(b), and 2(c) and Table 4).

### 3.3. Effects of BPS on Histopathology and Ultrastructure of the Kidney Injury in HFD/STZ-Induced Diabetic Rats

**Figure 3:**
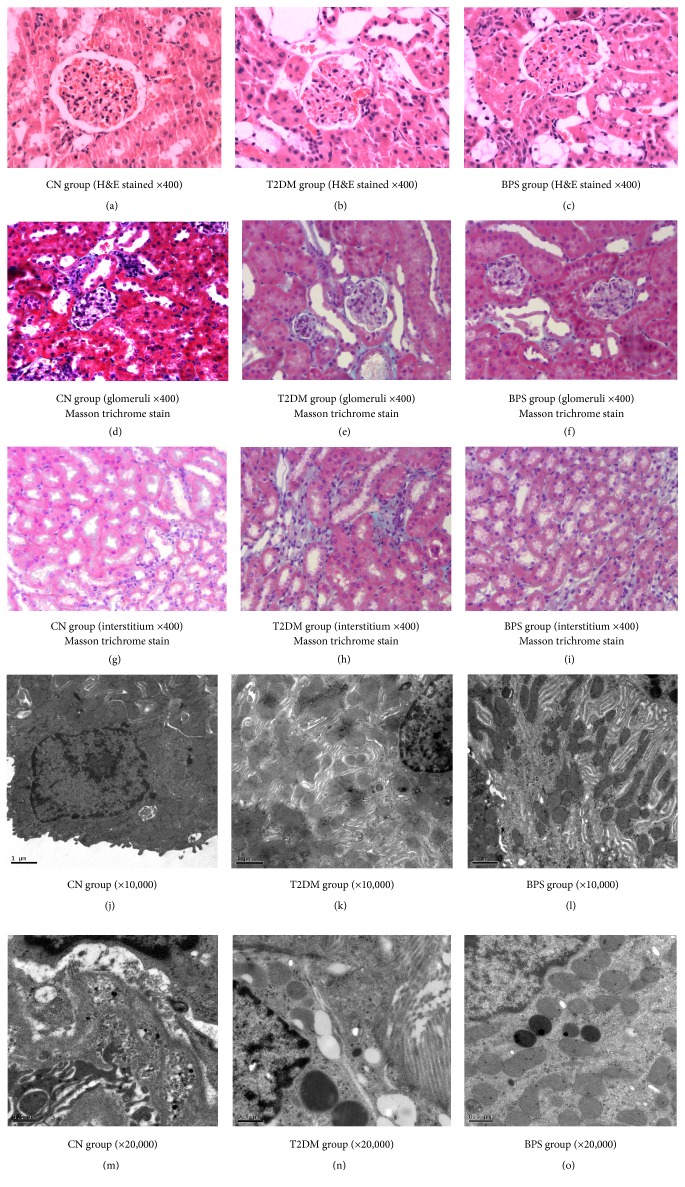
Effects of BPS on histopathology and ultrastructure of the kidney injury in HFD/STZ-induced diabetic rats.

Histological studies (H&E stained) on HFD/STZ-induced diabetic kidney of T2DM group showed increased glomerular size and exhibited focal and segmental glomerulosclerosis and expansion of glomerular matrix. Furthermore, tubular damage such as tubular atrophy, interstitial fibrosis, thickening of the tubular basement membranes, significant hydropic changes in the proximal convoluted tubules, and infiltration of inflammatory cells were also observed (Figures 3(b), 3(e), and 3(h)), compared with CN group (Figures 3(a), 3(d), and 3(g)). These alterations were effectively decreased on posttreatment with BPS for 12 weeks (Figures 3(c), 3(f), and 3(i)). These results again suggest the protective action of BPS in diabetic renal injury.

For electron micrograph, the renal cortex tubular tissues in CN group showed the normal cellular structure, abundant mitochondria, and intact nuclear membrane (Figures 3(j) and 3(m)). The ultrastructural changes occurred in the renal cortex tubular tissues of T2DM group (Figures 3(k) and 3(n)), including damaged nuclear membrane, large vacuoles in the cytoplasm, and swollen mitochondria. These changes were comparatively mild in kidney from rats in BPS group (Figures 3(l) and 3(o)), showing similar pattern of BPS in kidney protection, compared with rats in T2DM group.

### 3.4. Effects of BPS on HFD/STZ-Induced Inflammation Related Parameters

**Figure 4:**
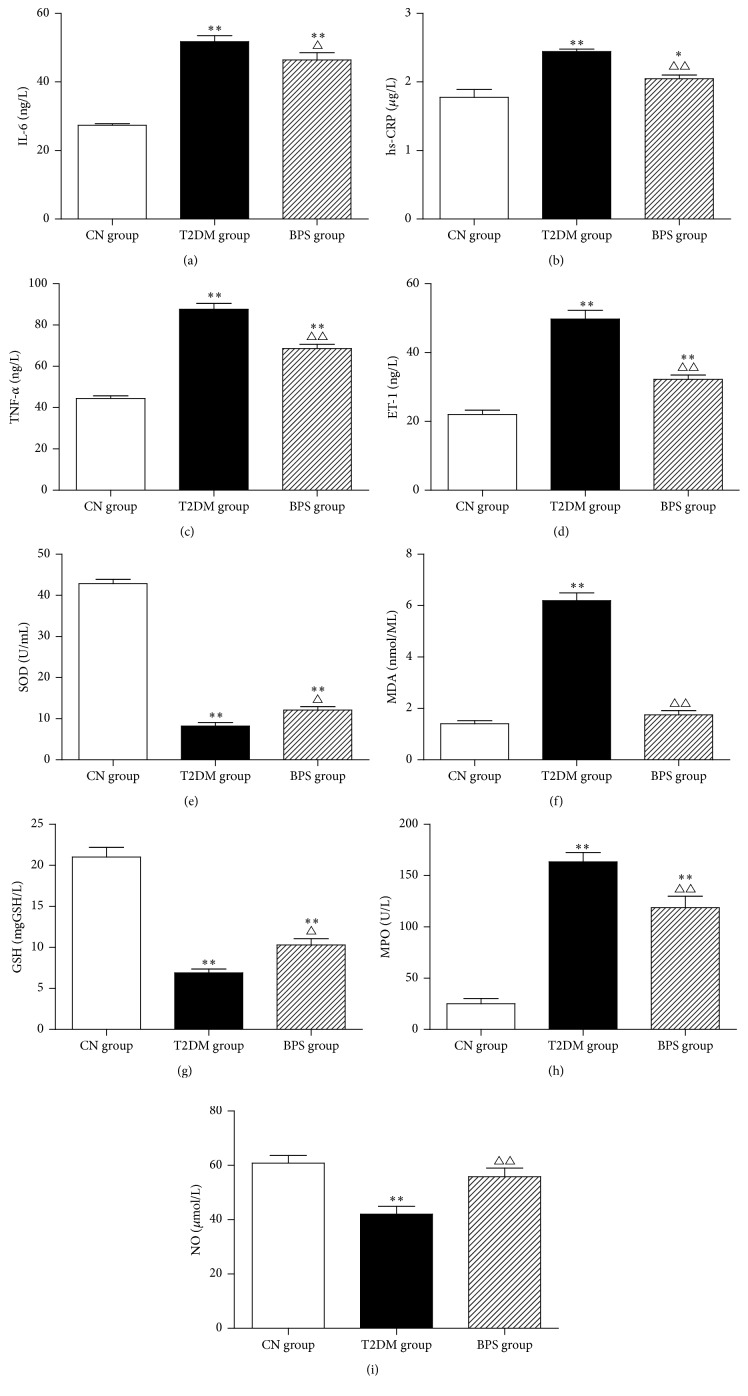
IL-6 (a), hs-CRP (b), TNF-*α* (c), ET-1 (d), SOD (e), MDA (f), GSH (g), MPO (h), and NO (i), in CN group rats, T2DM group rats, and BPS group rats. As bar graph, data are mean ± SD of 6 rats. ^*∗*^ *P* < 0.05 and ^*∗∗*^ *P* < 0.01 T2DM group and BPS group versus CN group rats and ^∆^ *P* < 0.05 and ^∆∆^ *P* < 0.01 BPS group versus T2DM group.

Compared with CN group, the level of IL-6, hs-CRP, TNF-*α*, ET-1, MDA, and MPO significantly increased in DM group. However, compared with DM group, the level of IL-6, hs-CRP, TNF-*α*, ET-1, MDA, and MPO effectively decreased in BPS group (Figures 4(a), 4(b), 4(c), 4(d), 4(f), and 4(h) and Table 5). The level of total SOD, GSH, and NO showed significant reduction in rats of T2DM group, compared with CN group. However, BPS treatment efficiently elevated the level of total SOD, GSH, and NO (Figures 4(e), 4(g), and 4(i) and Table 5). HFD/STZ-induced rats in T2DM group was characterized by the significant increase in inflammatory cytokines, inducing the damage of vascular endothelium and activation of oxidative stress. However, posttreatment with BPS for 12 weeks effectively decreased the alterations in these inflammation related parameters suggesting it to be a good anti-inflammatory agent that protects rat kidney from hyperglycemia-mediated inflammation.

### 3.5. Immunohistochemical Staining of CD31 and Collagen Type IV in Kidneys of the HFD/STZ-Induced Diabetic Rats

**Figure 5:**
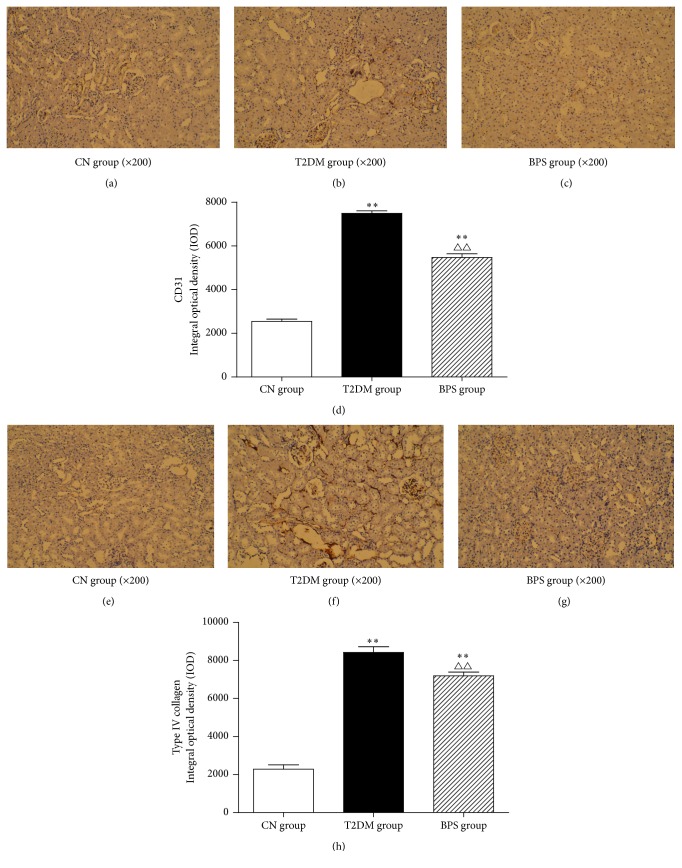
Immunohistochemical staining of CD31 (a, b, c) and collagen type IV (e, f, g) in Kidney tissues of CN group rats, T2DM group rats, and BPS group rats. As bar graph, data are mean ± SD of 6 rats. ^*∗*^ *P* < 0.05 and ^*∗∗*^ *P* < 0.01 T2DM group and BPS group versus CN group rats and ^∆^ *P* < 0.05 and ^∆∆^ *P* < 0.01 BPS group versus T2DM group.

The endothelium of the glomeruli strongly expressed CD31 in HFD/STZ-induced diabetic rats. Positive CD31 of immunohistochemical staining showed dark brown and CD31-positive area was significantly increased in T2DM group and BPS group. However, compared with rats in T2DM group, BPS treatment efficiently reduced immunohistochemical expression of CD31, and integral optical density (IOD) of CD31 significantly decreased (Figures 5(a), 5(b), 5(c), and 5(d) and Table 6).

Glomerular mesangial matrix strongly expressed type IV collagen in HFD/STZ-induced diabetic rats. Positive type IV collagen of immunohistochemical staining also showed dark brown, and the mesangial area of positive type IV collagen was significantly increased in T2DM group and BPS group. However, compared with rats in T2DM group, BPS treatment efficiently reduced immunohistochemical expression of type IV collagen, and integral optical density (IOD) of type IV collagen significantly decreased (Figures 5(e), 5(f), 5(g), and 5(h) and Table 6), suggesting that BPS can protect rats kidney from hyperglycemia-induced glomerular injury.

### 3.6. RT-PCR Results of p38 MAPK Gene in the Kidney Tissues of the HFD/STZ-Induced Diabetic Rats

**Figure 6:**
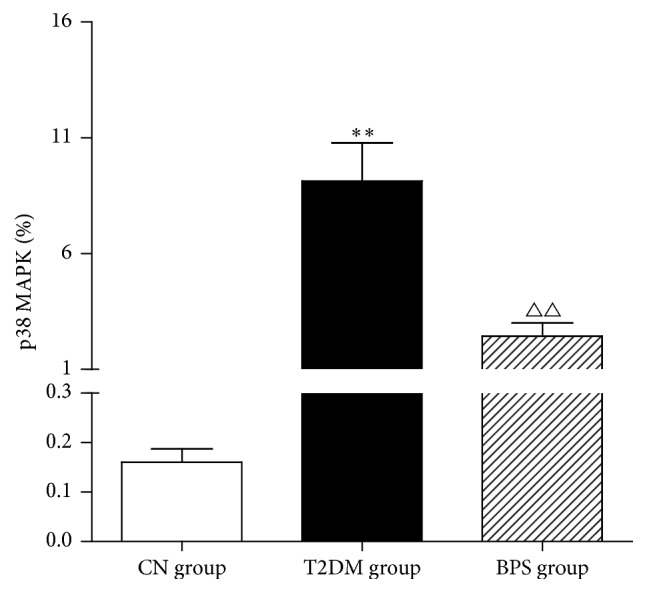
Comparison of p38 MAPK mRNA in kidney tissues of CN group rats, T2DM group rats, and BPS group rats. As bar graph, data are mean ± SD of 6 rats. ^*∗*^ *P* < 0.05 and ^*∗∗*^ *P* < 0.01 T2DM group and BPS group versus CN group rats and ^∆^ *P* < 0.05 and ^∆∆^ *P* < 0.01 BPS group versus T2DM group.

Compared with the CN group, the expression of p38 MAPK mRNA in the kidney tissues of rats in T2DM group was significantly higher. However, posttreatment with BPS in HFD/STZ-induced diabetic rats effectively reduced p38 MAPK mRNA level. The results suggest that BPS could efficiently prevent the expression of p38 MAPK mRNA in kidney of HFD/STZ-induced diabetic rats (Figure 6, Table 7).

### 3.7. Western Blot Results of p38 MAPK Signaling Pathway Related Protein and Inflammatory Factors

**Figure 7:**
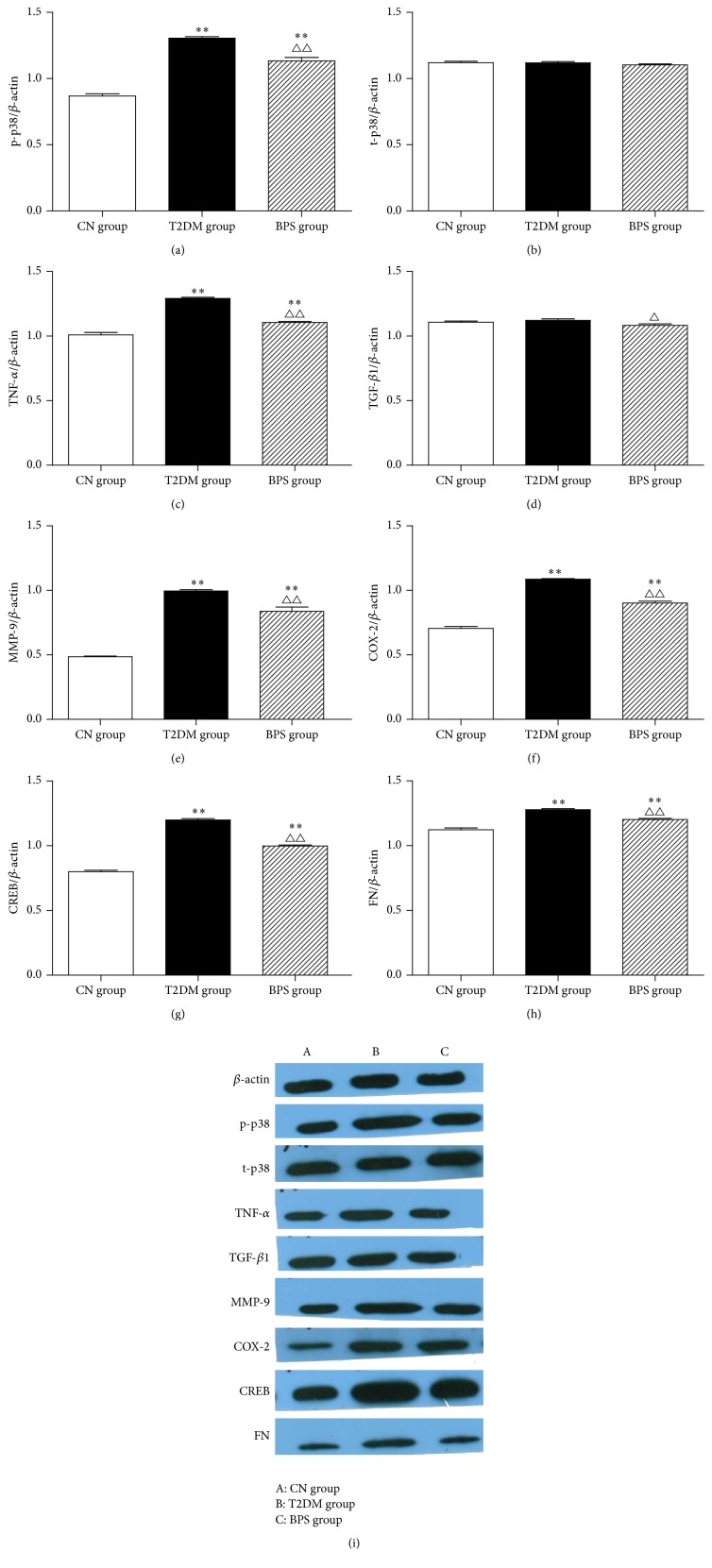
Western blot results of p-p38 (a), t-p38 (b), TNF-*α* (c), TGF-*β*1 (d), MMP-9 (e), COX-2 (f), CREB (g), and FN (h) in CN group rats, T2DM group rats, and BPS group rats. As bar graph, data are mean ± SD of 6 rats. ^*∗*^ *P* < 0.05 and ^*∗∗*^ *P* < 0.01 T2DM group and BPS group versus CN group rats and ^∆^ *P* < 0.05 and ^∆∆^ *P* < 0.01 BPS group versus T2DM group.

p38 MAPK plays a critical role in the regulation of inflammatory responses for the pathogenesis of diabetic nephropathy. To examine whether BPS can inhibit p38 MAPK signaling pathway in diabetic nephropathy, we performed a Western blot. In HFD/STZ-induced diabetic kidney tissues, expression of the phosphorylation of p38 MAPK increased, but expression of total p38 MAPK was unaffected. Compared with the T2DM group, BPS treatment could effectively decrease the expression of phosphorylation of p38 MAPK (Figures 7(a) and 7(b) and Table 8).

Inflammatory cytokines including TNF-*α*, MMP-9, and COX-2 lead the deterioration of diabetic nephropathy. In our study, HFD/STZ-induced diabetic rats showed significantly increased production of TNF-*α*, MMP-9, and COX-2 in the kidney tissue. On the other hand, compared with the T2DM group, BPS efficiently reduced the production of TNF-*α*, MMP-9, and COX-2 in the kidney tissue (Figures 7(c), 7(e), and 7(f), Table 8).

TGF-*β*1, FN, and CREB are important for the induction of fibrosis often associated with diabetic nephropathy. Western blot analysis showed that the expression levels of FN and CREB were significantly increased in the kidney tissue of HFD/STZ-induced diabetic rats and were efficiently inhibited by the posttreatment with BPS. However, the expression levels of TGF-*β*1 showed no significant differences in rats among the three groups (Figures 7(d), 7(g), and 7(h) and Table 8).

## 4. Discussion

Our present study established that BPS could provide protection against diabetic nephropathy of HFD/STZ-induced diabetic rats via the reversal of the activation of p38 MAPK signaling pathway and inhibiting inflammation involved in this pathophysiology. On the other hand, BPS could also improve metabolism of HFD/STZ-induced diabetic rats, reducing blood glucose and blood lipid. As we know, the pathology of type 2 diabetes is complex and establishing a suitable animal model of type 2 diabetes is important to investigate the pathogenesis of the disease.

Various studies have found that the HFD/STZ rat model might be a suitable animal model of type 2 diabetes, which would help us develop better therapeutics for type 2 diabetes [23–25]. In our study, we worked with the HFD/STZ rat as type 2 diabetic rat model of hyperglycemia, dyslipidemia, kidney dysfunction, and inflammation. Rats in T2DM group were characterized by increased plasma glucose level along with increased kidney-to-body weight ratio, indicating renal injury. A significant increase in plasma BUN, creatinine, and 24-hour urinary albumin also indicated the progressive nephrotoxicity in rats of T2DM group. Recent studies showed that BPS, a prostacyclin analog, could suppress the pathogenesis and development of diabetes and its complication by improving glucose intolerance, dyslipidemia, and insulin resistance [18, 26]. In our study, BPS effectively reversed this pathophysiology by lowering plasma BUN, creatinine, and 24-hour urinary albumin. BPS also reduced the plasma glucose level, restored kidney-to-body weight ratio, and altered hyperglycemia-induced inflammation.

The typical pathological changes in DN were mesangial cell proliferation, mesangial expansion accompanied by the accumulation of extracellular matrix and thickening of glomerular capillary walls, and fully developed diabetic glomerulopathy accompanied by nodular sclerosis [3]. Wang et al. [27] reported that the value of albuminuria excretion in STZ-induced diabetes rats was reduced by administration of BPS. They demonstrated that BPS corrected glomerular hyperfiltration and decreased albuminuria of early diabetic nephropathy. In this study, we demonstrated that BPS attenuated the severity of diabetic nephropathy in HFD/STZ-induced diabetic rats. There were significant differences in urinary protein, serum levels of BUN, and creatinine between rats in T2DM group and BPS group. Histological studies (H&E and Masson trichrome stained) on HFD/STZ-induced diabetic kidney of T2DM group showed increased glomerular size and exhibited focal and segmental glomerulosclerosis and expansion of glomerular matrix. Furthermore, tubular damage such as tubular atrophy, interstitial fibrosis, thickening of the tubular basement membranes, significant hydropic changes in the proximal convoluted tubules, and infiltration of inflammatory cells were also observed (Figures 3(b), 3(e), and 3(h)), compared with CN group (Figures 3(a), 3(d), and 3(g)). Renal hypertrophy was also evidenced by the increase in the weight of kidneys in T2DM group. These alterations were effectively decreased on posttreatment with BPS for 12 weeks (Figures 3(c), 3(f), and 3(i)). These histological alterations may result from the treatment of BPS, suggesting the protective action of BPS in diabetic renal injury. Some studies using electron microscopic morphometric analyzed the renal structural changes and the structural-functional relationships of diabetic nephropathy and found that mesangial expansion was a crucial structural change leading to loss of renal function in diabetes [28]. Electron microscopy revealed a spectrum of damage that included basement membrane thickening, loss of podocytic foot processes, and disruption of tubular basal infoldings and their related mitochondria and fibrosis of the tubules might be partly responsible for the clinical presentation of diabetic nephropathy [29]. In our study, the ultrastructural changes occurred in the renal cortex tubules of T2DM group (Figures 3(k) and 3(n)), including damaged nuclear membrane, large vacuoles in the cytoplasm, and swollen mitochondria. These changes were comparatively mild in kidney from rats in BPS group (Figures 3(l) and 3(o)), showing similar pattern of BPS in kidney protection, compared with rats in T2DM group.

Glomerular and interstitial fibrosis are the key morphological features of diabetic nephropathy (DN), and both correlate well with the development and progression of renal disease [30]. While mesangial cells and podocytes are thought to be major mediators of DN, increasing evidence suggests that renal tubulointerstitial fibrosis also plays a key role in the progression to end-stage renal disease, making this an important therapeutic target [31]. Interstitial renal fibrosis is characterized by tubular atrophy/dilation, interstitial leukocyte infiltration, accumulation of fibroblasts, and increased interstitial matrix deposition. CD31 is a marker of vascular endothelial cells. Some studies have shown that CD31 is positively expressed and highlights the glomerular endothelial cells from diabetic rats [32]. In our study, the endothelium of the glomeruli strongly expressed CD31 in HFD/STZ-induced diabetic rats. Positive CD31 of immunohistochemical staining showed dark brown, and expressing the endothelial marker protein CD31 was much reduced in glomerular endothelium in rats of BPS group compared to those of T2DM group (Figures 5(a), 5(b), 5(c), and 5(d) and Table 6). These observations suggested that altered glomerular and interstitial fibrosis might take place in treatment of BPS.

Researchers have found that increased glomerular and mesangial deposition of collagen IV occurs in diabetic nephropathy and increases the extent of renal injury [33]. In our study, treatment with BPS, a stable prostaglandin I_2_ analogue, prevented the stimulation by high glucose of the increased secretion and biosynthesis of collagen IV. Compared with rats in T2DM group, BPS treatment efficiently reduced immunohistochemical expression of type IV collagen, and integral optical density (IOD) of type IV collagen significantly decreased (Figures 5(e), 5(f), 5(g), and 5(h) and Table 6), suggesting that BPS can protect rats kidney from hyperglycemia-induced glomerular injury.

Elevated blood levels of inflammatory markers have been associated with increased risk for DN onset and progression. C-reactive protein (CRP), an acute phase reactant, is associated with all-cause mortality in patients with end-stage renal disease (ESRD). TNF-*α* and IL-6, key cytokines that mediate both acute and chronic inflammation, are associated with all-cause morbidity and mortality in the general population and in predialysis and dialysis patients. Some study findings suggest that TNF-*α*, IL-6, and CRP are associated with the prevalence and severity of DN [34]. In patients with DN, inflammation leads to endothelial dysfunction, increased circulating levels of endothelin-1 (ET-1) [35]. Some studies also show that the high concentration of MPO is a diagnostically significant parameter in the prediction of endothelial dysfunction in patients with T2DM [36]. Increased level of MDA is an index of endogenous lipid peroxidation; induction of diabetes significantly increased MDA levels, reflecting inflammation and oxidative stress, especially in patients with DN [37]. The recent studies find that low-grade inflammation may negatively affect glycemic control and *β*-cell function in the early time course of type 1 and type 2 diabetes. Increased inflammation will also influence the progression of diabetes and the occurrence of macro- and microvascular diabetes-related complications at later stages of the disease [38]. As we know, inflammation relates to activation of oxidative stress and may contribute to accelerated progression of DN. Oxidative stress-related biomarkers, such as SOD, GSH, and NO, are linked to inflammation in kidney in patients with DN [39]. In our study, we examined whether elevated levels of inflammation factors, activation of oxidative stress, and endothelial dysfunction in kidney, involved in the pathogenesis of DN, could be ameliorated by treatment with BPS. The level of IL-6, hs-CRP, TNF-*α*, ET-1, MDA, and MPO significantly increased in DM group. However, compared with DM group, the level of IL-6, hs-CRP, TNF-*α*, ET-1, MDA, and MPO effectively decreased in BPS group (Figures 4(a), 4(b), 4(c), 4(d), 4(f), and 4(h) and Table 5), suggesting that BPS exerts anti-inflammatory effects. The level of total SOD, GSH, and NO showed significant reduction in rats of T2DM group. However, BPS treatment efficiently elevated the level of total SOD, GSH, and NO (Figures 4(e), 4(g), and 4(i) and Table 5), showing significant antioxidative stress activity. HFD/STZ-induced rats in T2DM group were characterized by the significant increase in inflammatory cytokines, inducing the damage of vascular endothelium and activation of oxidative stress. Posttreatment with BPS for 12 weeks effectively decreased the alterations in these inflammation related parameters, suggesting it to be a good anti-inflammatory agent that protects rat kidney from hyperglycemia-mediated inflammation.

The p38 MAPK has been implicated in a wide range of complex biologic processes, such as cell proliferation, cell differentiation, cell death, cell migration, and invasion [40]. p38 MAPK signaling pathway is activated through the double phosphorylation of p38 MAPK into p-p38 MAPK in response to diverse stimuli and mediates its function by components downstream of inflammatory factors [9]. The activation of p38 MAPK, one of the downstream effectors of inflammation and fibrosis, has been reported to be involved in the progression of DN [41]. Dysregulation of p38 MAPK levels is associated with advanced stages in patients with DN. In diabetic animal models, p38 MAPK activity rapidly increases in glomeruli and tubules after the induction of hyperglycemia and is also found in the accumulating kidney interstitial cells associated with advanced nephropathy [42]. Studies of nondiabetic kidney disease have shown that pharmacological inhibition of p38 MAPK suppressed inflammation and fibrosis [43]. The object of this study is to determine the pathogenic role of p38 MAPK signalling in the progression of DN and the role of p38 MAPK as an attractive target with intervention of BPS. Our study showed that the expression of p38 MAPK mRNA in the kidney tissues of rats in T2DM group was significantly higher. However, posttreatment with BPS in HFD/STZ-induced diabetic rats effectively reduced p38 MAPK mRNA level. The results suggest that BPS could efficiently prevent the expression of p38 MAPK mRNA in kidney of HFD/STZ-induced diabetic rats (Figure 6, Table 7). Western blot results showed that, in HFD/STZ-induced diabetic kidney tissues, expression of the phosphorylation of p38 MAPK increased but expression of total p38 MAPK was unaffected. Compared with the T2DM group, BPS treatment could effectively decrease the expression of phosphorylation of p38 MAPK (Figures 7(a) and 7(b) and Table 8). The results suggested that the p38 MAPK signaling pathway was activated in the T2DM rat model, inflammatory factors increased, and kidney injury accelerated. BPS protected the kidney against inflammation by downregulating the activity of the p38 MAPK signaling pathway.

The p38 MAPK is activated in kidney tissues by inflammatory cytokines such as TNF-*α*, MMP-9, and COX-2, leading to the deterioration of DN. TNF-*α* is mainly produced by monocytes, macrophages and T cells but also intrinsic kidney cells [38]. Experimental studies in animal models of diabetes have showed that TNF-*α* protein and expression levels are enhanced in renal glomeruli and tubules. TNF-*α* may cause direct cytotoxicity to renal cells, inducing direct renal injury [44]. Recent studies show that TNF-*α* upregulate MMP-9 expression. MMP-9 both is the end product of inflammation and acts as an inflammatory mediator that participates in inflammatory reactions and tissue destruction. MMP-9 plays a key role in the development of DN [45]. Overexpression of endogenous MMP-9 induced podocyte dedifferentiation. MMP-9 also interrupted podocyte cell integrity and promoted podocyte monolayer permeability to albumin and extracellular matrix protein synthesis. In diabetic patients, the upregulation of urinary MMP-9 concentrations occurred earlier than the onset of microalbuminuria [46]. COX-2 is an enzyme that is found in several different tissues in the body. COX-2 appears to produce a substance called prostaglandins, mainly at sites of inflammation [47]. Kidneys of patients with DN overexpress COX-2 in various regions. Prostaglandins, which are produced in the kidney by COX-2, may contribute to glomerular and tubulointerstitial inflammatory diseases (types of kidney diseases due to inflammation). BPS is a stable, orally active prostacyclin analogue with vasodilatory, antiplatelet, and cytoprotective effects, acting similar to COX-2 inhibitors. In our study, BPS showed potentially beneficial effects in reducing the amount of protein spilled in the urine and preserving kidney function in the T2DM rat model by inhibition of inflammation. HFD/STZ-induced diabetic rats showed significantly increased production of TNF-*α*, MMP-9, and COX-2 in the kidney tissue. On the other hand, compared with the T2DM group, BPS efficiently reduced the production of TNF-*α*, MMP-9, and COX-2 in the kidney tissue (Figures 7(c), 7(e), and 7(f) and Table 8).

TGF-*β*1 is an ubiquitously expressed cytokine belonging to a large superfamily of activins/bone morphogenetic proteins [48]. This mediator plays an active role in the processes of proliferation, wound healing, synthesis of ECM molecules, and fibrotic reaction [49]. TGF-*β*1, therefore, strongly contributes to fibrotic disorders such as diabetic nephropathy. TGF-*β*1 is particularly important in the mediation of expansion and later fibrosis via the stimulation of collagen and FN [50]. TGF-*β*1 activates CREB protein in a PKA dependent manner. Decreasing cAMP accompanied by PKA reduced CREB phosphorylation accelerates renal tubulointerstitial fibrosis. Our Western blot analysis showed that the expression levels of FN and CREB were significantly increased in the kidney tissue of HFD/STZ-induced diabetic rats and were efficiently inhibited by the posttreatment with BPS, suggesting that BPS inhibited the activation of p38 MAPK signaling pathway and attenuated the upregulated fibrosis process in this pathophysiological condition of DN (Figures 7(g) and 7(h) and Table 8).

**Figure 8:**
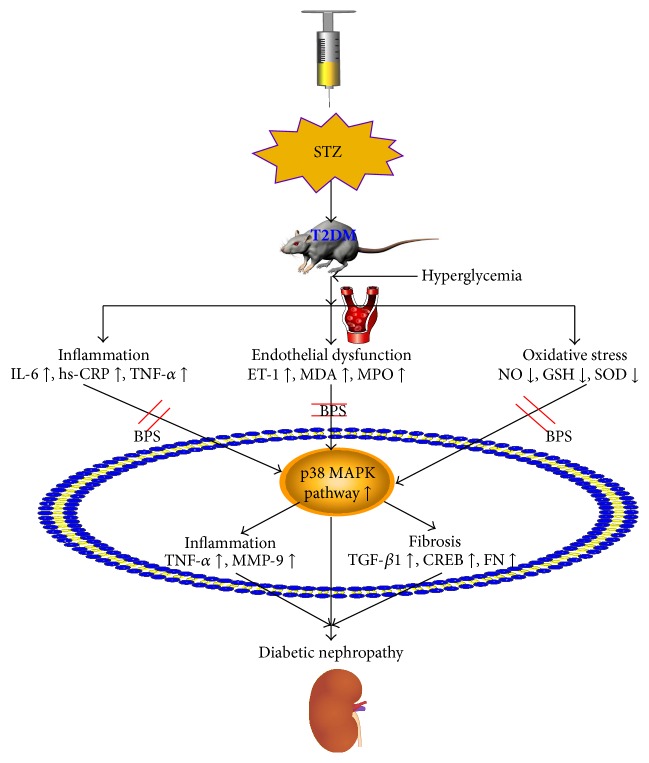
Protection mechanisms of BPS in diabetic nephropathy.

In conclusion, the current results suggest that BPS improved kidney function and urinary albumin excretion through modulating p38 MAPK signaling pathway in HFD/STZ-induced diabetic rats, a typical T2DM animal model. Moreover, BPS ameliorated glomerular expression of CD31 and type IV collagen. The protective mechanisms are complicated but may be mainly attributed to the inhibition inflammation and fibrosis in the diabetic kidney by downregulation of the p38 MAPK signaling pathway. BPS treatment could provide effective protection against inflammation, endothelial dysfunction, and oxidative injury in the renal tissue of STZ induced type 2 diabetic rats via p38 MAPK signaling pathway, ameliorating the process of inflammation and fibrosis in kidney tissue (Figure 8). Overall, BPS may be beneficial for the therapy in patients with DN. The relationship between different subtypes of p38 MAPK and development of DN also requires further studies.

## Figures and Tables

**Table 1:** Primer sequences.

Primer	Sequence (5′-3′)	Annealing temperature (°C)
p38 MAPK	Forward primer: 5′-TTCCCAGCAGTCCTATCC-3′	55
	Reverse primer: 5′-GTCAGATGGCAAGGGTTC-3′	

GAPDH	Forward primer: 5′-TTGCTGATGACTGGTTACAATACA-3′	55
	Reverse primer: 5′-GCTTGACTTACAGAAGAATCGTTG-3′	

**Table 2:** Changes in body weight of rats in experiment (*n* = 6, $\bar{X}\pm S$).

Group	0 weeks (g)	4 weeks (g)	8 weeks (g)	12 weeks (g)
CN group	186.47 ± 14.29	324.38 ± 41.20	407.03 ± 41.00	454.38 ± 32.87
T2DM group	187.43 ± 13.86	403.40 ± 31.19^*∗∗*^	354.27 ± 47.44^*∗*^	352.47 ± 36.18^*∗∗*^
BPS group	184.10 ± 13.53	413.65 ± 24.04^*∗∗*^	394.42 ± 30.94	426.80 ± 42.97^△△^

^*∗*^ *P* < 0.05 and ^*∗∗*^ *P* < 0.01 T2DM group and BPS group compared with the CN group; ^△^ *P* < 0.05 and ^△△^ *P* < 0.01 BPS group compared with the T2DM group.

**Table 3:** Body weight, kidney weight, kidney weight/body weight, blood glucose, and blood lipid profile (TC, TG, HDL, and LDL) of rats in experiment (*n* = 6, $\bar{X}\pm S$).

	CN group	T2DM group	BPS group
Body weight (g)	454.38 ± 32.87	352.47 ± 36.18^*∗∗*^	426.80 ± 42.97^△△^
Kidney weight (mg)	1462.79 ± 127.51	2087.78 ± 223.08^*∗∗*^	2148.41 ± 517.99^*∗∗*^
Kidney weight/body weight (g/mg)	3.22 ± 0.22	5.93 ± 0.38^*∗∗*^	4.99 ± 0.91^*∗∗*△^
Blood glucose (mmol/L)	6.1 ± 0.43	23.75 ± 1.75^*∗∗*^	17.96 ± 3.19^*∗∗*△△^
TC (mmol/L)	2.22 ± 0.17	9.78 ± 0.84^*∗∗*^	8.75 ± 0.82^*∗∗*△^
TG (mmol/L)	0.63 ± 0.07	3.99 ± 0.31^*∗∗*^	3.58 ± 0.52^*∗∗*^
LDL-C (mmol/L)	0.31 ± 0.16	4.88 ± 1.20^*∗∗*^	3.53 ± 0.63^*∗∗*△△^
HDL-C (mmol/L)	1.71 ± 0.10	0.68 ± 0.24^*∗∗*^	1.19 ± 0.56^*∗*△^

^*∗*^ *P* < 0.05 and ^*∗∗*^ *P* < 0.01 T2DM group and BPS group compared with CN group rats and ^△^ *P* < 0.05 and ^△△^ *P* < 0.01 BPS group compared with the T2DM group.

**Table 4:** Kidney function and 24 h urinary albumin of rats in experiment (*n* = 6, $\bar{X}\pm S$).

	CN group	T2DM group	BPS group
Plasma creatinine (*μ*mol/L)	47.11 ± 14.62	87.41 ± 19.41^*∗∗*^	65.92 ± 14.79^△^
Plasma BUN (mmol/L)	12.17 ± 2.19	26.09 ± 3.26^*∗∗*^	24.99 ± 2.95^*∗∗*^
24 h urinary albumin (mg)	4.24 ± 1.15	16.59 ± 1.69^*∗∗*^	13.89 ± 0.99^*∗∗*△△^

^*∗*^ *P* < 0.05 and ^*∗∗*^ *P* < 0.01 T2DM group and BPS group compared with CN group rats and ^△^ *P* < 0.05 and ^△△^ *P* < 0.01 BPS group compared with the T2DM group.

**Table 5:** HFD/STZ-induced inflammation-related parameters of rats in experiment (*n* = 6, $\bar{X}\pm S$).

	CN group	T2DM group	BPS group
IL-6 (ng/L)	27.39 ± 1.12	51.80 ± 4.23^*∗∗*^	46.43 ± 5.11^*∗∗*△^
hs-CRP (*μ*g/L)	1.78 ± 0.28	2.44 ± 0.09^*∗∗*^	2.05 ± 0.14^*∗*△△^
TNF-*α* (ng/L)	44.42 ± 3.17	87.65 ± 6.85^*∗∗*^	68.59 ± 4.90^*∗∗*△△^
ET-1 (ng/L)	22.05 ± 2.98	49.72 ± 6.20^*∗∗*^	32.21 ± 3.15^*∗∗*△△^
SOD (*μ*/ml)	42.85 ± 2.57	8.24 ± 2.16^*∗∗*^	12.11 ± 2.03^*∗∗*△^
MDA (nmol/L)	1.40 ± 0.29	6.20 ± 0.73^*∗∗*^	1.75 ± 0.40^△△^
GSH (mgGSH/L)	21.01 ± 2.93	6.90 ± 1.16^*∗∗*^	10.31 ± 1.80^*∗∗*△^
MPO (*μ*/L)	25.07 ± 12.64	163.35 ± 22.62^*∗∗*^	118.73 ± 27.55^*∗∗*△△^
NO (*μ*mol/L)	60.81 ± 6.88	42.00 ± 7.03^*∗∗*^	55.82 ± 7.89^△△^

^*∗*^ *P* < 0.05 and ^*∗∗*^ *P* < 0.01 T2DM group and BPS group compared with CN group rats and ^△^ *P* < 0.05 and ^△△^ *P* < 0.01 BPS group compared with the T2DM group.

**Table 6:** Immunohistochemical expression of CD31 and collagen type IV in kidney tissues of rats in experiment (*n* = 6, $\bar{X}\pm S$).

Integrated optical density (IOD)	CN group	T2DM group	BPS group
CD31	2546.83 ± 253.05	7485.00 ± 289.74^*∗∗*^	5467.50 ± 422.93^*∗∗*△△^
Collagen type IV	2283.67 ± 554.55	8418.00 ± 738.00^*∗∗*^	7185.50 ± 482.97^*∗∗*△△^

^*∗*^ *P* < 0.05 and ^*∗∗*^ *P* < 0.01 T2DM group and BPS group compared with CN group rats and ^△^ *P* < 0.05 and ^△△^ *P* < 0.01 BPS group compared with the T2DM group.

**Table 7:** p38 MAPK mRNA in the kidney tissues of rats in experiment using RT-PCR (*n* = 6, $\bar{X}\pm S$).

Group	*N*	Expression of p38 MAPK mRNA (%)
CN group	6	0.1599 ± 0.0663
DM group	6	9.1258 ± 4.0717^*∗∗*^
BPS group	6	2.4334 ± 1.3923^△△^

^*∗*^ *P* < 0.05 and ^*∗∗*^ *P* < 0.01 T2DM group and BPS group compared with CN group rats and ^△^ *P* < 0.05 and ^△△^ *P* < 0.01 BPS group compared with the T2DM group.

**Table 8:** Western blot results of p-p38, t-p38, TNF-*α*, TGF-*β*1, MMP-9, COX-2, CREB, and FN in kidney tissues of rats in experiment (*n* = 6, $\bar{X}\pm S$).

Optical density ratios	CN group	T2DM group	BPS group
p-p38 MAPK/*β*-actin	0.8683 ± 0.0388	1.3051 ± 0.2737^*∗∗*^	1.1326 ± 0.0663^*∗∗*△△^
t-p38 MAPK/*β*-actin	1.1202 ± 0.0263	1.1196 ± 0.0228	1.1029 ± 0.0184
TNF-*α*/*β*-actin	1.010 ± 0.0458	1.2913 ± 0.0219^*∗∗*^	1.1060 ± 0.0220^*∗∗*△△^
TGF-*β*1/*β*-actin	1.1074 ± 0.0225	1.1215 ± 0.2794	1.0832 ± 0.2881^△^
MMP-9/*β*-actin	0.4862 ± 0.0121	0.9954 ± 0.0258^*∗∗*^	0.8375 ± 0.0796^*∗∗*△△^
COX-2/*β*-actin	0.7056 ± 0.0373	1.087 ± 0.0105^*∗∗*^	0.9017 ± 0.03756^*∗∗*△△^
CREB/*β*-actin	0.7996 ± 0.0258	1.199 ± 0.0238^*∗∗*^	0.9986 ± 0.0163^*∗∗*△△^
FN/*β*-actin	1.1235 ± 0.0339	1.2778 ± 0.0226^*∗∗*^	1.2017 ± 0.0224^*∗∗*△△^

^*∗*^ *P* < 0.05 and ^*∗∗*^ *P* < 0.01 T2DM group and BPS group compared with CN group rats and ^△^ *P* < 0.05 and ^△△^ *P* < 0.01 BPS group compared with the T2DM group.
